# Dose–Response Relationships of Resistance Training in Healthy Old Adults: A Systematic Review and Meta-Analysis

**DOI:** 10.1007/s40279-015-0385-9

**Published:** 2015-09-29

**Authors:** Ron Borde, Tibor Hortobágyi, Urs Granacher

**Affiliations:** Division of Training and Movement Sciences, Research Focus Cognition Sciences, University of Potsdam, Am Neuen Palais 10, Building 12, 14469 Potsdam, Germany; Center for Human Movement Sciences, University Medical Center Groningen, University of Groningen, Groningen, The Netherlands; Faculty of Health and Life Sciences, Northumbria University, Newcastle Upon Tyne, UK

## Abstract

**Background:**

Resistance training (RT) is an intervention frequently used to improve muscle strength and morphology in old age. However, evidence-based, dose–response relationships regarding specific RT variables (e.g., training period, frequency, intensity, volume) are unclear in healthy old adults.

**Objectives:**

The aims of this systematic review and meta-analysis were to determine the general effects of RT on measures of muscle strength and morphology and to provide dose–response relationships of RT variables through an analysis of randomized controlled trials (RCTs) that could improve muscle strength and morphology in healthy old adults.

**Data Sources:**

A computerized, systematic literature search was performed in the electronic databases PubMed, Web of Science, and The Cochrane Library from January 1984 up to June 2015 to identify all RCTs related to RT in healthy old adults.

**Study Eligibility Criteria:**

The initial search identified 506 studies, with a final yield of 25 studies. Only RCTs that examined the effects of RT in adults with a mean age of 65 and older were included. The 25 studies quantified at least one measure of muscle strength or morphology and sufficiently described training variables (e.g., training period, frequency, volume, intensity).

**Study Appraisal and Synthesis Methods:**

We quantified the overall effects of RT on measures of muscle strength and morphology by computing weighted between-subject standardized mean differences (SMD_bs_) between intervention and control groups. We analyzed the data for the main outcomes of one-repetition maximum (1RM), maximum voluntary contraction under isometric conditions (MVC), and muscle morphology (i.e., cross-sectional area or volume or thickness of muscles) and assessed the methodological study quality by Physiotherapy Evidence Database (PEDro) scale. Heterogeneity between studies was assessed using *I*^2^ and *χ*^2^ statistics. A random effects meta-regression was calculated to explain the influence of key training variables on the effectiveness of RT in terms of muscle strength and morphology. For meta-regression, training variables were divided into the following subcategories: volume, intensity, and rest. In addition to meta-regression, dose–response relationships were calculated independently for single training variables (e.g., training frequency).

**Results:**

RT improved muscle strength substantially (mean SMD_bs_ = 1.57; 25 studies), but had small effects on measures of muscle morphology (mean SMD_bs_ = 0.42; nine studies). Specifically, RT produced large effects in both 1RM of upper (mean SMD_bs_ = 1.61; 11 studies) and lower (mean SMD_bs_ = 1.76; 19 studies) extremities and a medium effect in MVC of lower (mean SMD_bs_ = 0.76; four studies) extremities. Results of the meta-regression revealed that the variables “training period” (*p* = 0.04) and “intensity” (*p* < 0.01) as well as “total time under tension” (*p* < 0.01) had significant effects on muscle strength, with the largest effect sizes for the longest training periods (mean SMD_bs_ = 2.34; 50–53 weeks), intensities of 70–79 % of the 1RM (mean SMD_bs_ = 1.89), and total time under tension of 6.0 s (mean SMD_bs_ = 3.61). A tendency towards significance was found for rest in between sets (*p* = 0.06), with 60 s showing the largest effect on muscle strength (mean SMD_bs_ = 4.68; two studies). We also determined the independent effects of the remaining training variables on muscle strength. The following independently computed training variables are most effective in improving measures of muscle strength: a training frequency of two sessions per week (mean SMD_bs_ = 2.13), a training volume of two to three sets per exercise (mean SMD_bs_ = 2.99), seven to nine repetitions per set (mean SMD_bs_ = 1.98), and a rest of 4.0 s between repetitions (SMD_bs_ = 3.72). With regard to measures of muscle morphology, the small number of identified studies allowed us to calculate meta-regression for the subcategory training volume only. No single training volume variable significantly predicted RT effects on measures of muscle morphology. Additional training variables were independently computed to detect the largest effect for the single training variable. A training period of 50–53 weeks, a training frequency of three sessions per week, a training volume of two to three sets per exercise, seven to nine repetitions per set, a training intensity from 51 to 69 % of the 1RM, a total time under tension of 6.0 s, a rest of 120 s between sets, and a rest of 2.5 s between repetitions turned out to be most effective.

**Limitations:**

The current results must be interpreted with caution because of the poor overall methodological study quality (mean PEDro score 4.6 points) and the considerable large heterogeneity (*I*^2^ = 80 %, *χ*^2^ = 163.1, *df* = 32, *p* < 0.01) for muscle strength. In terms of muscle morphology, our search identified nine studies only, which is why we consider our findings preliminary. While we were able to determine a dose–response relationship based on specific individual training variables with respect to muscle strength and morphology, it was not possible to ascertain any potential interactions between these variables. We recognize the limitation that the results may not represent one general dose–response relationship.

**Conclusions:**

This systematic literature review and meta-analysis confirmed the effectiveness of RT on specific measures of upper and lower extremity muscle strength and muscle morphology in healthy old adults. In addition, we were able to extract dose–response relationships for key training variables (i.e., volume, intensity, rest), informing clinicians and practitioners to design effective RTs for muscle strength and morphology. Training period, intensity, time under tension, and rest in between sets play an important role in improving muscle strength and morphology and should be implemented in exercise training programs targeting healthy old adults. Still, further research is needed to reveal optimal dose–response relationships following RT in healthy as well as mobility limited and/or frail old adults.

## Key Points

**Table:** 

Meta-regression of data from 25 studies revealed that a resistance training (RT) program with the goal to increase healthy old adults’ muscle strength is characterized by a training period of 50–53 weeks, a training intensity of 70–79 % of the one-repetition maximum (1RM), a time under tension of 6 s per repetition, and a rest in between sets of 60 s. Selecting a training frequency of two sessions per week, a training volume of two to three sets per exercise, seven to nine repetitions per set, and a rest of 4.0 s between repetitions could also improve efficacy of training.
The meta-regression revealed that none of the examined training variables of volume (e.g., period, frequency, number of sets, number of repetitions) predicted the effects of RT on measures of muscle morphology. Yet, RT to improve muscle morphology seems to be effective using the following independently computed training variables: a training period of 50–53 weeks, a training frequency of three sessions per week, a training volume of two to three sets per exercise, seven to nine repetitions per set, a training intensity from 51 to 69 % of the 1RM, a total time under tension of 6.0 s, a rest of 120 s between sets, and a 2.5-s rest between repetitions.
This meta-analysis provides preliminary data for therapists, practitioners, and clinicians regarding relevant RT variables and their dose–response relationships to improve muscle strength and morphology in healthy old adults.

## Introduction

With the onset of the sixth decade in life, degenerative processes affect the neuromuscular system in terms of losses in muscle strength (dynapenia) and muscle mass (sarcopenia) [1–3]. Neural (e.g., numerical loss of alpha motoneurons) and morphological factors (e.g., reduced number and size of particularly type-II muscle fibers) as well as their interaction are responsible for age-related declines in muscle strength and mass [4]. There is evidence that muscular weakness is highly associated with impaired mobility and an increased risk for falls [5]. Moreover, lower extremity muscle weakness was identified as the dominant intrinsic fall-risk factor with a five-fold increase in risk of falling [5]. Although the age-related decline in muscle strength is associated with the loss in muscle size (*r* = 0.66–0.83, *p* < 0.001) [6], longitudinal studies found a 1.5 to five times greater decline in muscle strength compared with muscle size [2, 7]. In addition, there was a stronger relationship between muscle strength and physical performance or disability compared with the relationship between muscle strength and mass [3].

Even though exercise cannot fully prevent aging of the neuromuscular system, resistance training (RT) has a great potential to mitigate age-related changes. Over the past 25–30 years, numerous studies have examined the effects of RT on measures of muscle strength and morphology in old adults. Frontera and Bigard [8] reviewed RT’s potential to improve old adults’ muscle strength and morphology [6]. The review highlighted two studies that examined (a) the impact of aging on muscle strength (i.e., maximal isokinetic knee extensor torque) and muscle size [i.e., cross-sectional area (CSA) of the knee extensors] in elderly men with a mean age of 65 years, followed over a 12-year period [7], and (b) the effects of a 12-week RT program (three sessions/week) on the same variables of muscle strength and size in a cohort of 60- to 72-year-old men [9]. Findings from the 12-year longitudinal study revealed a loss in isokinetic knee extensor torque of −24 % and in quadriceps CSA of −16 %. In contrast, 12 weeks of RT at 80 % of the one-repetition maximum (1RM) resulted in an increase in isokinetic torque of 16 % and in knee extensor CSA of 11 %. Even though different cohorts were investigated in the two studies, the reported percentage rates are impressive and may allow a cautious and preliminary conclusion that biological aging of the neuromuscular system can be mitigated or even reversed to a certain extent [8].

Relying on an extensive database comprising individual experimental studies and reviews, the American College of Sports Medicine (ACSM) issued what is considered as the gold standard of RT exercise prescription for healthy old adults [10]. However, a careful examination of this position stand suggests that the position stand was based on category 4 or ‘expert level’ evidence on the evidence pyramid, the lowest compared with evidence level 1 provided by systematic reviews and meta-analyses [11]. Considering that the already published meta-analyses are methodologically limited in terms of study selection criteria {inclusion of non-randomized controlled trials (RCTs) [12, 13] }, the number of included training variables (e.g., traditional variables such as training period, frequency, volume, intensity only) [14–16], and by focusing only on direct comparisons of intervention groups (e.g., high- vs. low-intensity) [14], it seems imperative and timely to quantify the dose–response relationships through a systematic review and meta-analysis. To the best of our knowledge, a meta-analysis that only includes RCTs and is based on a comparison between an intervention group and a physically inactive control group is currently missing in the literature. In contrast to direct comparisons (high- vs. low-intensity intervention groups), we investigate the effects of RT in sedentary older adults when starting RT compared with physically inactive control groups to mitigate the age-related loss of muscle strength and morphology. A review of existing data concerning so far overlooked variables such as time under tension and rest time would more comprehensively inform clinicians and practitioners on how to standardize RT. Finally, potential influences of the included training variables on the investigated effects of RT on muscle strength and morphology will be examined using meta-regression. Meta-regression will be performed for relevant subcategories of training variables (i.e., volume, intensity, rest). Thus, the purpose of the present systematic review and meta-analysis is to determine the general effects of RT on measures of muscle strength and morphology. Furthermore, the present meta-analysis, using meta-regression, examines how specific training variables affect muscle strength and morphology. We constructed dose–response relationships for key RT variables [17] through the analysis of RCTs that have clearly improved measures of muscle strength and morphology in healthy old adults.

## Methods

The present meta-analysis follows the recommendations of the ‘Preferred Reporting Items for Systematic Reviews and Meta-Analyses’ (PRISMA) [18].

### Search Strategy

A systematic literature search was conducted from January 1984 to June 2015 in the online databases PubMed, Web of Science, and The Cochrane Library. The following Medical Subject Headings (MeSH) of the United States National Library of Medicine (NLM) and search terms were included in our Boolean search syntax: (“resistance training” OR “strength training” OR “weight training” OR “weight-bearing exercise program”) AND (old* OR elderly) AND (sarcopenia OR dynapenia OR “muscle strength” OR “muscle morphology”). The search was limited to English language, human species, age 65+ years, full text availability, and RCTs.

### Selection Criteria/Study Eligibility

Inclusion criteria were decided by the consensus statements of two reviewers (RB, UG). In cases where RB and UG did not reach agreement on inclusion of an article, TH was contacted. In accordance with the PICOS approach [18], inclusion criteria were selected by (a) population: healthy subjects who were aged ≥60 years, with a study mean age ≥65 years; (b) intervention: machine-based RT containing a description of at least one training variable (e.g., training intensity); (c) comparator: non-physically active (e.g., health education, no intervention) control groups; (d) outcome: at least one proxy of muscle strength [e.g., 1RM, maximum voluntary contraction under isometric conditions (MVC)] and/or muscle morphology [e.g., CSA (cm^2^, mm), volume (kg, cm^3^), thickness (mm)]; and (e) study design: RCTs [18]. Studies were excluded if they (a) did not meet the minimum requirements regarding the description of training variables (e.g., period, frequency, volume, intensity); (b) tested multiple repetition maximum (e.g., 3RM); (c) did not report results adequately (mean and standard deviation); (d) included frail, mobility and/or cognitively limited and/or ill subjects; (e) examined the effects of concurrent training (i.e., combined RT and endurance training); and (f) investigated the effects of nutritional supplements in combination with RT. If multiple outcomes (e.g., strength properties of different muscle groups) were recorded within one study, we chose the outcome with the highest functional relevance for mobility in old age. In other words, (a) lower extremity muscle strength tests were preferred over upper extremity muscle strength tests; (b) isokinetic or dynamic muscle strength tests were preferred over isometric tests; and (c) multi-joint tests (e.g., leg press) were chosen rather than single-joint strength tests (e.g., leg extension/curl). In terms of muscle groups, sub-analyses were computed for muscles of upper and lower extremities. Tests for the assessment of muscle strength were analyzed separately for the 1RM and MVC. Measures of muscle morphology were included if one of the following devices was used: magnetic resonance imaging, computed tomography, dual x-ray absorptiometry, ultrasound, or BOD POD (air displacement plethysmograph for whole-body densitometry). In addition, one representative part of the respective muscle (e.g., vastus lateralis) had to be assessed either by muscle CSA, volume, or thickness when more than one muscle was tested.

### Coding of Studies

The studies were coded for the following variables: (a) cohort; (b) age; (c) training variables [i.e., period, frequency, volume (i.e., number of sets per exercise, number of repetitions per set), intensity, time under tension (total, isometric, concentric, eccentric), and rest (rest in between sets and repetitions)]; (d) strength tests (i.e., 1RM, MVC); (e) body region (i.e., upper limbs, lower limbs); and (f) assessment of muscle morphology (i.e., CSA, muscle volume, muscle thickness). The RT groups were subdivided according to the applied training intensity: high-intensity RT: ≥70 % 1RM; moderate-intensity RT: 51 % ≥ 1RM ≤ 69 %; and low-intensity RT: ≤50 % 1RM [16]. In the dose–response relationship figures presented in the “Results” section, diamonds, circles, and triangles symbolize high- (≥70 % 1RM), moderate- (51 % ≥ 1RM ≤ 69 %), and low- (≤50 % 1RM) intensity RT groups. If exercise progression was realized over the course of the intervention or if training variables were reported, the average of these variables was calculated. If results of pre- and post-tests were not conclusively reported, the authors of the respective studies were contacted via email. Six out of 12 authors responded to our queries and subsequently sent the missing data to calculate SMD_bs_.

### Data Extraction

The main study characteristics (i.e., cohort, age, intervention program, training variables, relevant outcomes) were extracted in an Excel template/spreadsheet.

### Assessment of Methodological Study Quality

Evaluation of methodological study quality was conducted by two independent reviewers using the Physiotherapy Evidence Database (PEDro) scale [19]. The PEDro scale includes 11 items with three items from the Jadad scale [20] and nine items from the Delphi list [21]. PEDro rates RCTs on a scale from 0 (low quality) to 10 (high quality), with a score of ≥6 representing a cut-off for high-quality studies [19]. The first item of the PEDro scale (eligibility criteria were specified) is used to establish external validity and is therefore not included in the overall score. Maher et al. [19] demonstrated fair-to-good inter-rater reliability, with an intra-class correlation coefficient of 0.68 when using consensus ratings generated by two or three independent raters.

### Statistical Analyses

To determine overall effects of RT on measures of muscle strength and morphology and to establish dose–response relationships following RT in old adults, the between-subject standardized mean differences (SMD_bs_) were calculated according to the following formula: ${\text{SMD}}_{i} = \frac{{m_{1i} - m_{2i} }}{{s_{i} }}$ [22], where SMD_*i*_ is the standardized mean difference of one reported parameter (e.g., strength properties of quadriceps muscle), *m*_1*i*_ and *m*_2*i*_ correspond to the mean of the intervention and the control groups, respectively and *s*_*i*_ is the pooled standard deviation. In accordance with Hedges and Olkin, this formula was adjusted for sample size: $g = \left( {1 - \frac{3}{{4N_{i} - 9}}} \right)$ [23], where *N*_*i*_ is the total sample size of the intervention group and control group. SMD_bs_ is defined as the difference between the post-test treatment and the control means divided by the pooled standard deviation, with 95 % confidence intervals (CIs). If two or more studies reported the same training variable (e.g., training volume, intensity, rest), weighted mean SMD_bs_ over the studies was calculated and presented as filled squares in the dose–response relationship figures presented in the Sect. 3. Each unfilled symbol illustrates SMD_bs_ per single training group. Within-subject standardized mean difference (SMD_ws_) was calculated as follows: ±(mean of post-test − mean of pre-test)/SD pre-value, where SD is the standard deviation. Positive SMD values indicate a favorable effect of RT as compared with the control condition. Our meta-analysis was conducted using Review Manager version 5.3.4 (Copenhagen: The Nordic Cochrane Centre, The Cochrane Collaboration, 2008). The included studies were weighted by the standard error: ${\text{SE}} \left\{ {{\text{SMD}}_{i} } \right\} = \sqrt {\frac{{N_{i} }}{{n_{1i} n_{2i} }} + \frac{{SMD_{i}^{2} }}{{2(N_{i - 3.94)} }}}$ [22], where *n*_1*i*_ is the sample size of the intervention group and *n*_2*i*_ is the sample size of the control group. Given that variability (e.g., different age and muscle groups) between studies was large, we decided to compute a random-effects model to estimate the effects of RT interventions [18, 24]. According to Cohen, effect size values of 0.00 to ≤0.49 indicate small, values of 0.50 to ≤0.79 indicate medium, and values ≥0.80 indicate large effects [25]. Heterogeneity was assessed using *I*^2^ and *χ*^2^ statistics. Furthermore, a random effects meta-regression was performed to examine whether the effects of RT on measures of muscle strength and morphology are predicted according to the combined values of the different training variables using the valid software Comprehensive Meta-analysis version 3.3.070 (Biostat Inc., NJ, USA) [26–28]. Subcategories were created to extract the most important training variables of the following combinations: training volume (i.e., period, frequency, number of sets per exercise, number of repetitions per set); training intensity (i.e., intensity, time under tension) and rest (rest in between sets and repetitions) [29, 30]. For each subcategory, random-effects meta-regression was performed to identify variables that best predict the differences in the effect sizes of improvements in measures of muscle strength and morphology. According to Toigo and Boutellier [17], RT variables were previously reported insufficiently in the literature. Thus, we decided to report dose–response relationships of each RT variable that could maximize improvements in measures of muscle strength and morphology [17].

## Results

**Fig. 1:**
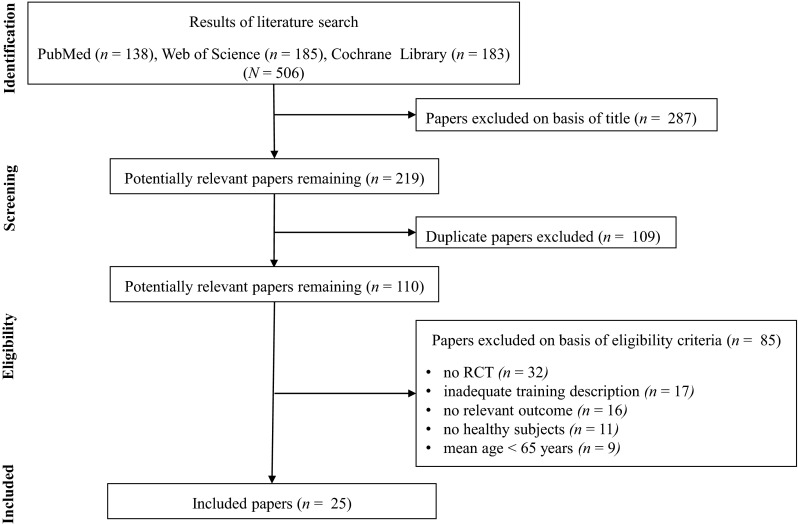
Flow chart presenting the different steps of search and study selection. *RCT* randomized controlled trial

Our systematic literature search identified 506 potentially relevant studies (Fig. 1). A screening of the titles excluded 287 studies and then 109 duplicates were removed. The remaining 110 studies were analyzed concerning the pre-defined eligibility criteria, and 85 of these were removed. Finally, 25 studies with a total of 819 participants (mean sample size 33 subjects) and a mean age of 70.4 years (age range 60–90 years) were included in the quantitative synthesis (Table 1). Furthermore, four out of 25 studies investigated the effects of high-intensity RT compared with low-intensity RT (i.e., ≤50 % 1RM) [31–34]. Three studies [31, 33, 35] analyzed the effects of high-intensity RT compared with RT at moderate intensities (i.e., 51 % ≥ 1RM ≤ 69 %).

**Table 1:** Studies examining the effects of RT on variables of muscle strength and muscle morphology in healthy old adults

Study	Sex	Age (years)	*N*	Muscles/functional movement	Period (weeks)	Strength gain (%)	Gain in measure of muscle morphology (%)	Within subject SMD (SMD_ws_)	Between subject SMD (SMD_bs_)	Training variables
Beneka et al. [31]	M/F	66–72 Mean age: 69	M: 8/8/8/8 HI/MI/LI/CG F: 8/8/8/8 HI/MI/LI/CG	Knee extension	16	1RM male HI: 11 MI: 8 LI: 4 CG: −2 n.s. 1RM female HI: 15 MI: 7 LI: 3 CG: −1 n.s.		1RM male HI: 1.36 MI: 1.14 LI: 0.43 CG: −0.16 1RM female HI: 3.58 MI: 0.71 LI: 0.69 CG: −0.13	1RM male HI vs. CG: 1.17 MI vs. CG: 0.77 LI vs. CG: 0.25 HI vs. MI: 0.33 HI vs. LI: 1.03 MI vs. LI: 0.60 1RM female HI vs. CG: 1.92 MI vs. CG: 0.62 LI vs. CG: 0.83 n.s. HI vs. MI: 3.18 HI vs. LI: 3.49 MI vs. LI: −0.10	RT: 3×/week; 3 sets HI: 4–6 reps; 90 % 1RM MI: 8–10 reps; 70 % 1RM LI: 12–14 reps; 50 % 1RM; TUT: 6 s; 2 s con, 2–3 s iso, 2–3 s ecc; RIS: 120 s; RIR: 5 s; weight machines CG: no intervention
Charette et al. [92]	F	64–86 Mean age: 68	13/6	Leg press	12	1RM RT: 27–106 CG: −2 to 11 n.s.		1RM RT: 5.92–11.00 CG: −0.12 to 1.17	1RM RT vs. CG: 1.98–7.42	RT: 3×/week; 3–6 sets; 6 reps; 1–5 weeks: 65 % 1RM 6–9 weeks: 70 % 1RM 10–12 weeks: 75 % 1RM; TUT: 5 s; 2 s con, 3 s ecc; weight machines CG: no intervention
Daly et al. [93]	M/F	Mean age: 75	8/8	Upper extremity	6	1RM RT: −33 to 14 NPA CG: −19 to 28 NPA	MRI/MV RT: 1–4 NPA CG: −3 to −1 NPA	1RM RT: −0.07 to 1.00 CG: −0.41 to 0.11 MV RT: −0.11 to 0.13 CG: −0.02 to −0.08	1RM RT vs. CG: −0.17 to 0.50 MV RT vs. CG: 0.36–0.52	RT: 3×/week; 1 week: 3 sets; 8 reps; 60 % 1RM 2 weeks: 3 sets; 8 reps; 70 % 1RM 3–6 weeks: 2 sets; 8 reps; 75 % 1RM; RIS: 60–90 s; weight machines and free weight CG: no intervention
DeBeliso et al. [94]	M/F	63–83 Mean age: 72	13/17/13 FR/PER/CG	Lower extremity	18	1RM FR: 50–67 PER: 70–81 CG: −5 to 25		1RM FR: 1.40–2.33 PER: 1.08–2.09 CG: −0.10 to 0.72	1RM FR vs. CG: 1.33–1.80 PER vs. CG: 1.22–1.37 FR vs. PER: 0.07–0.21	RT: 2×/week; FR: 3 sets; 9RM PER: 1–6 weeks; 2 sets; 15RM 7–12 weeks; 3 sets; 9RM 13–18 weeks; 4 sets; 6RM; 60 min; RIS: 120–180 s; weight machines CG: no intervention
Fatouros et al. [95]	M	65–78 Mean age: 70	8/8	Upper/lower extremity	16	IS RT: 14 CG: −1 n.s. 1RM upper RT: 114 CG: 1 n.s. 1RM lower RT: 77 CG: 3 n.s.		IS RT: 1.71 CG: −0.08 1RM upper RT: 6.65 CG: 0.02 1RM lower RT: 7.23 CG: 0.20	IS RT vs. CG: 1.38 1RM upper RT vs. CG: 3.65 1RM lower RT vs. CG: 4.88	RT: 3×/week; 1–4 weeks: 2 sets; 13 reps; 55–60 % 1RM 5–8 weeks: 3 sets; 12 reps; 60–70 % 1RM 9–12 weeks: 3 sets; 10 reps; 70–80 % 1RM 13–16 weeks; 3 sets; 8 reps; 80 % 1RM; 45–50 min; TUT: 7.5 s; 2–3 s con, 2 s iso, 2–3 s ecc; RIS: 120 s; RIR: 5 s; weight machines CG: no intervention
Fatouros et al. [33]	M	65–78 Mean age: 71	14/12/14/10 HI/MI/LI/CG	Upper/lower extremities	24	1RM upper HI: 73 MI: 48 LI: 34 CG: 2 n.s. 1RM lower HI: 63 MI: 53 LI: 38 CG: −2 n.s.		1RM upper HI: 3.52 MI: 2.25 LI: 1.77 C: 0.10 1RM lower HI: 4.94 MI: 5.45 LI: 4.86 C: −0.18	1RM upper HI vs. CG: 2.71 MI vs. CG: 1.93 LI vs. CG: 1.38 HI vs. MI: 0.78 HI vs. LI: 1.44 MI vs. LI: 0.63 1RM lower HI vs. CG: 4.10 MI vs. CG: 3.75 LI vs. CG: 3.34 HI vs. MI: 0.62 HI vs. LI: 1.81 MI vs. LI: 1.22	RT: 3×/week; 2–3 sets; 8–15 reps HI: 80 % 1RM MI: 60 % 1RM LI: 40 % 1RM; TUT: 7.5 s; 2–3 s con, 2–3 s iso, 2–3 s ecc; HI RIS: 360 s MI RIS: 240 s LI RIS: 120 s; RIR: 3–5 s; weight machines CG: no intervention
Granacher et al. [36]	M/F	60–80 Mean age: 67	20/20	Lower extremity	13	MVC RT: 27 CG: −4 n.s.		MVC RT: 1.24 CG: −0.16	MVC RT vs. CG: 1.15	RT: 3×/week; 3 sets; 10 reps; 80 % 1 RM; 60-min sessions; RIS: 120 s; weight machines; CG: no intervention
Henwood and Taaffe [40]	M/F	65–84 Mean age: 70	22/22	Upper/lower extremities	8	1RM upper RT: 2 n.s. –25 CG: −3 to −14 n.s. 1RM lower RT: 11–27 CG: −10 to 3 n.s.		1RM upper RT: 0.06–0.54 CG: −0.30 to −0.09 1RM lower RT: 0.35–1.06 CG: −0.22 to 0.07	1RM upper RT vs. CG: 3.62–5.02 1RM lower RT vs. CG: 4.30–7.66	RT: 2×/week; 3 sets; 8 reps; 75 % 1RM; 60-min sessions; RIS: 60 s; TUT: 6 s; con: 3 s, ecc: 3 s; weight machines CG: no intervention
Hortobagyi et al. [34]	M/F	66–83 Mean age: 72	9/9/9 HI/LI/CG	Leg press	10	MVC HI: 24 n.s. LI: 28 n.s. CG: 2 n.s. IS HI: 38 n.s. LI: 29 n.s. CG: 1 n.s. 1RM HI: 35 n.s. LI: 33 n.s. CG: 3 n.s.		MVC HI: 1.06 LI: 1.00 CG: −0.10 IS HI: 1.17 LI: 0.84 CG: −0.02 1RM HI: 1.05 LI: 0.78 CG: −0.10	MVC HI vs. CG: 0.89 LI vs. CG: 0.67 HI vs. LI: 0.03 n.s. IS HI vs. CG: 0.86 LI vs. CG: 0.37 HI vs. LI: 0.45 n.s. 1RM HI vs. CG: 1.05 LI vs. CG: 0.52 HI vs. LI: 0.41 n.s.	RT: 3 ×/week; HI: 5 sets; 4–6 reps; 80 % 1RM LI: 5 sets; 8–12 reps; 40 % 1RM; TUT: 3 s; 1–2 s con, 1–2 s ecc; RIS: 120 s; weight machines CG: no intervention
Hunter et al. [96]	M/F	61–77 Mean age: 66	14/14/14 HI/VI/CG	Knee extension/elbow flexion	25	1RM HI: 13–24 VI: 10–28 CG: −6 to −2 n.s.	BP/FFM HI: 4 VI: 4 CG: 1 n.s.	1RM HI: 0.43–0.74 VI: 0.21–0.75 CG: −0.18 to −0.04 FFM HI: 0.19 VI: 0.17 CG: 0.03	1RM HI vs. CG: 0.85–1.13 VI vs. CG: 0.05–0.67 HI vs. VI: 0.61–0.96 n.s. FFM HI vs. CG: 0.38 VI vs. CG: −0.23 HI vs. CG: 0.71 n.s.	RT: 3 ×/week; 2 sets; 10 reps; 45-min session; RIS: 120 s; weight machines HI: 80 % 1RM VI: 50, 65, 80 % 1RM across the 3 sessions per week CG: no intervention
Judge et al. [43]	M/F	≥75 Mean age: 80	28/27	Lower extremity	13	1RM RT: 12 CG: −3 n.s.		1RM RT: 0.64 CG: −0.05	1RM RT vs. CG: 0.11	RT: 3×/week; 3 sets; 12 reps; 75 % RM; 45-min session; TUT: 4 s; 2 s con, 2 s ecc; RIS: 120–180 s; RIR: 1–2 s; weight machines CG: no intervention
Kalapotharakos et al. [35]	M/F	60–74 Mean age: 65	11/12/10 HI/MI/CG	Upper/lower extremities	12	1RM upper HI: 66 MI: 43 CG: −1 n.s. 1RM lower HI: 78 MI: 44 CG: 0 n.s	CT/CSA HI: 10 MI: 7 CG: −1 n.s.	1RM upper HI: 2.73 MI: 1.62 CG: −0.04 1RM lower HI: 3.13 MI: 1.45 CG: 0.02 CSA HI: 0.34 MI: 0.37 CG: −0.02	1RM upper HI vs. CG: 2.11 MI vs. CG: 1.47 HI vs. MI: 0.50 1RM lower HI vs. CG: 2.51 MI vs. CG: 1.51 HI vs. MI: 0.97 CSA HI vs. CG: 0.38 MI vs. CG: 0.34 HI vs. MI: 0.10	RT: 3×/week; 3 sets; HI: 8 reps; 80 % 1RM MI: 15 reps; 60 % 1RM; TUT: 6 s; 2 s con, 2 s iso, 2 s ecc; RIS: 120 s; RIR: 2–3 s; weight machines CG: no intervention
Kalapotharakos et al. [71]	M	61–75 Mean age: 68	9/9	Lower extremity	10	1RM RT: 24 CG: 0 n.s.		1RM RT: 0.83 CG: 0.01	1RM RT vs. CG: 1.50	RT: 3×/week; 3 sets; 15 reps; 60 % 1RM; 60-min session; RIS: 120 s; weight machines CG: no intervention
Lovell et al. [97]	M/F	70–80 Mean age: 74	12/12	Leg extension	16	1RM RT: 90 CG: −1 n.s.	CT/LM RT: 7 CG: 1 n.s.	1RM RT: 5.97 CG: −0.07 LM RT: 0.14 CG: 0.03	1RM RT vs. CG: 4.33 LM RT vs. CG: 0.10	RT: 3×/week; 3 sets; 6–10 reps; 70–90 % 1RM; RIS: 120 s; weight machines CG: no intervention
Miszko et al. [98]	M/F	65–90 Mean age: 72	13/15	Lower extremity	16	1RM upper RT: 14 CG: −1 n.s. 1RM lower RT: 23 CG: 5 n.s.		1RM upper RT: 0.28 CG: 0.01 1RM lower RT: 0.43 CG: 0.11	1RM upper RT vs. CG: 0.33 1RM lower RT vs. CG: 0.53	RT: 3×/week; 3 sets; 6–8 reps; 1–8 weeks: 50–70 % 1RM 9–16 weeks: 80 % 1RM; TUT: 4 s; 4 s con; weight machines + free weights CG: no intervention
Morse et al. [99]	M	70–82 Mean age: 74	13/8	Lower extremity (ankle)	52	MVC RT: 0 n.s.−25 CG: −2 to 5 n.s.	MRI/MV RT: 15 CG: 2 n.s.	MVC RT: 0.00–1.29 CG: −0.09 to 0.35 MV RT: 1.53 CG: 0.22	MVC RT vs. CG: 0.89 BD−1.51 MV RT vs. CG: 1.03	RT: 3×/week (2 × group based, 1 × home based); 2 − 3 sets; 8 − 10 reps; 80 % 1RM; rubber bands, weight machines CG: no intervention
Pinto et al. [41]	F	60–69 Mean age: 66	19/17	Lower extremity	6	1RM RT: 22 CG: −1 n.s.	US/MT RT: 11–21 CG: −5 to 7 n.s.	1RM RT: 1.16 CG: −0.04 MT RT: 0.59–0.90 CG: −0.38 to 0.24	1RM RT vs. CG: 1.33 MT RT vs. CG: 0.52–0.99	RT: 2×/week; 1–3 weeks: 2 sets; 15–20 reps 4–6 weeks: 3 sets; 12–15 reps; RIS: 120 s CG: no intervention
Pyka et al. [39]	M/F	61–78 Mean age: 68	8/6	Upper/lower extremities	52	1RM upper RT: 23–51 CG: −4 to −12 n.s. 1RM lower RT: 27–62 CG: −3 to −12 n.s.		1RM upper RT: 3.30–5.38 CG: −1.35 to −0.63 1RM lower RT: 4.50–9.51 CG: −1.45 to −0.32	1RM upper RT vs. CG: 4.69–6.12 1RM lower RT vs. CG: 5.87–7.67	RT: 3×/week; 3 sets; 8 reps; 65–75 % 1RM; 60-min sessions; TUT: 5 s; 2 s con, 3 s ecc; RIS: 60 s; weight machines CG: no intervention
Raso et al. [42]	F	60–77 Mean age: 68	14/9	Trunk/lower extremity	52	1RM RT: 48 CG: 5 n.s.	N/A/FFM RT: −3 n.s CG: −2 n.s.	1RM RT: 4.73 CG: 0.67 FFM RT: −0.22 CG: −0.20	1RM RT vs. CG: 2.20 FFM RT vs. CG: 0.20	RT: 3×/week; 3 sets; 12 reps; 55 % 1RM; 60-min sessions; TUT: 4 s; 1–2 s con, 2–3 s ecc; RIS: 120 s; weight machines CG: no intervention
Reeves et al. [37]	M/F	65–79 Mean age: 71	9/9	Lower extremity	14	MVC RT: 15 CG: −12 n.s.		MVC RT: 0.32 CG: −0.45	MVC RT vs. CG: 0.52 NPA	RT: 3×/week; 2 sets; 10 reps; 70–75 % 1RM; TUT: 5 s; 2 s con, 3 s ecc; RIS: 180 s; weight machines CG: no intervention
Rhodes et al. [100]	F	65–75 Mean age: 69	20/18	Upper/lower extremity	52	1RM upper RT: 9 n.s. –25 CG: 0–2 n.s. 1RM lower RT: 19–54 CG: −4 to 1 n.s.		1RM upper RT: 0.55–1.70 CG: 0.02–0.09 1RM lower RT: 0.83–2.62 CG: −0.21 to 0.06	1RM upper RT vs. CG: 0.60–1.25 1RM lower RT vs. CG: 1.28–2.85	RT: 3 ×/week; 3 sets; 8 reps; 75 % 1RM; 60-min sessions; TUT: 6 s; 2–3 s con, 3–4 s ecc CG: no intervention
Strasser et al. [72]	M/F	≥70 Mean age: 74	15/14	Upper/lower extremities	26	1RM upper RT: 24–31 CG: 3 n.s. 1RM lower RT: 15 CG: 9 n.s.		1RM upper RT: 0.61–0.76 CG: 0.10–0.12 1RM lower RT: 0.47 CG: 0.35	1RM upper RT vs. CG: 1.00–1.40 1RM lower RT vs. CG: 0.77 BD	RT: 3×/week; 3–6 sets; 10–15 reps; 60–70 % 1RM CG: no intervention
Tracy et al. [38]	M/F	65–80 Mean age: 74	11/9	Knee extension	16	MVC RT: 26 CG: −1 n.s. 1RM RT: 27 CG: 2 n.s.		MVC RT: 0.81 CG: −0.05 1RM RT: 0.67 CG: 0.05	MVC RT vs. CG: 0.27 1RM RT vs. CG: 0.53	RT: 3×/week; 3 sets; 10 reps; 80 % 1RM CG: no intervention
Vincent et al. [32]	M/F	60–83 Mean age: 68	22/24/16 HI/LI/CG	Upper/lower extremities, trunk (total strength)	24	1RM HI: 18 LI: 17 CG: −1 n.s.	CT/FFM HI: 0.4 n.s. LI: −3.6 n.s. CG: −1 n.s.	1RM HI: 0.42 LI: 0.45 CG: −0.04 FFM HI: 0.02 LI: −0.12 CG: −0.05	1RM HI vs. CG: 0.66 LI vs. CG: 0.49 HI vs. LI: 0.25 n.s. FFM HI vs. C: 0.17 LI vs. C: 0.22 HI vs. LI: −0.06 NPA	RT: 3×/week; 1 set; HI: 8 reps; 80 % 1RM LI: 13 reps; 50 % 1RM; RIS: 120 s; weight machines CG: no intervention
Vincent et al. [73]	M/F	60–72 Mean age: 69	10/10	Total body strength	24	1RM RT: 16 CG: −2 n.s.	CT/FFM RT: 4 n.s. CG: 1 n.s.	1RM RT: 1.35 CG: −0.15 FFM RT: 0.57 CG: 0.13	1RM RT vs. CG: 0.08 FFM RT vs. CG: 1.30	RT: 3×/week; 1 set; 8–13 reps; 50–80 % 1RM; weight machines CG: no intervention

*1RM* one-repetition maximum, *BD* baseline differences (*p* > 0.05), *BP* BOD POD (air displacement plethysmograph for whole-body densitometry), *CG* control group, *con* concentric, *CSA* cross-sectional area, *CT* computed tomography, *ecc* eccentric, *F* female, *FFM* fat-free mass, *FR* fixed repetitions, *HI* high-intensity, *IS* isokinetic strength, *iso* isometric, *LI* low-intensity, *LM* lean mass, *M* male, *MI* moderate-intensity, *MRI* magnetic resonance imaging, *MT* muscle thickness, *MV* muscle volume, *MVC* maximal voluntary contraction, *N/A* not available, *NPA* no *p* values available, *n.s.* not significant, *PER* periodized repetitions, *reps* repetitions, *RIR* rest in between repetitions, *RIS* rest in between sets, *RT* resistance training, *SMD*_*bs*_ difference between the post-test treatment and the control means divided by the pooled standard deviation with 95 % confidence intervals, *SMD*_*ws*_ difference of mean of post-test and mean of pre-test divided by standard deviation of pre-value, *TUT* total time under tension, *US* ultrasonography, *VI* variable intensity

### Overall Findings

#### Effects of Resistance Training (RT) on Measures of Muscle Strength

**Fig. 2:**
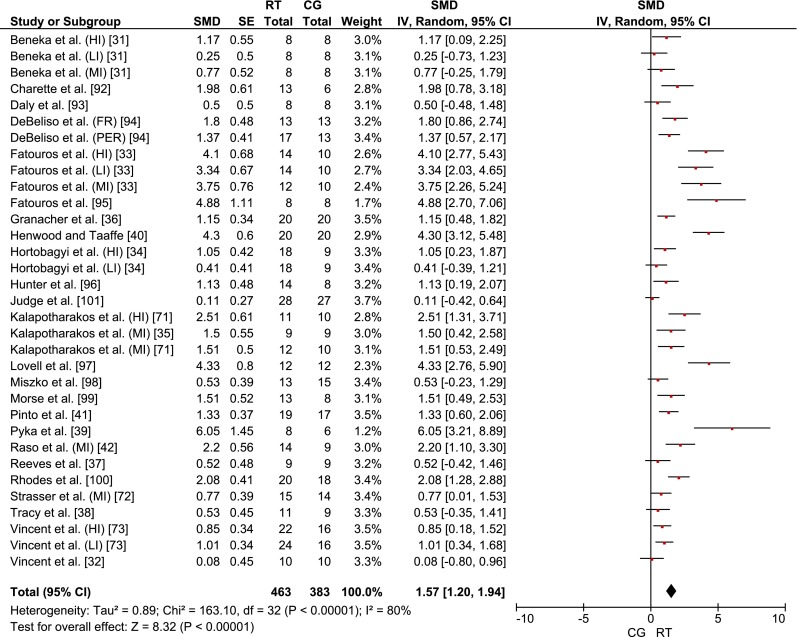
Effects of RT on measures of muscle strength. *CG* control group, *CI* confidence interval, *FR* fixed repetition training group, *HI* high-intensity training group, *IV* inverse variance, *LI* low-intensity training group, *MI* moderate-intensity training group, *PER* periodized repetition training group, *Random* random effects model, *RT* resistance training, *SE* standard error, *SMD* standardized mean difference, *Weight* weight attributed to each study due to its statistical power

All 25 studies reported a favorable effect of RT on upper and lower extremity muscle strength. Weighted mean SMD_bs_ for the effects of RT on muscle strength amounted to mean SMD_bs_ = 1.57 (95 % CI 1.20–1.94; *I*^2^ = 80 %, *χ*^2^ = 163.10, *df* = 32, *p* < 0.01) (Fig. 2), which is indicative of a large effect. In addition, in sub-analyses, we determined the effects of RT on upper and lower body strength tested by the 1RM. The analyses revealed weighted mean SMD_bs_ for the upper (mean SMD_bs_ = 1.61; 95 % CI 0.95–2.27; *I*^2^ = 86 %, *χ*^2^ = 88.52, *df* = 12, *p* < 0.01) and lower extremities (mean SMD_bs_ = 1.76; 95 % CI 1.20–2.31; *I*^2^ = 87 %, *χ*^2^ = 144.47, *df* = 19, *p* < 0.01), corresponding to large effects. There were no studies that tested MVC in upper extremity muscles. Only four studies measured leg muscle MVCs [34, 36–38]. A medium effect (mean SMD_bs_ = 0.76; 95 % CI 0.40–1.31) was found for MVC of lower limbs, with non-significant heterogeneity (*I*^2^ = 0 %, *χ*^2^ = 2.89, *df* = 4, *p* = 0.58).

#### Effects of RT on Measures of Muscle Morphology

**Fig. 3:**
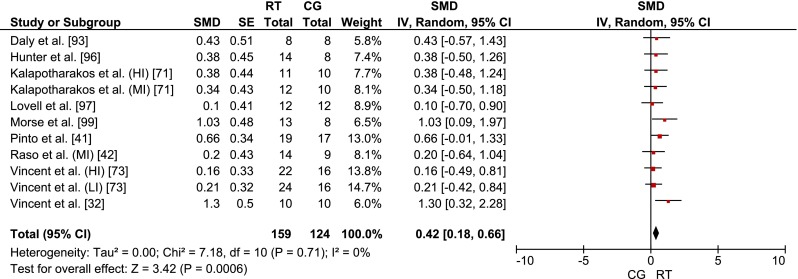
Effects of RT on measures of muscle morphology. *CG* control group, *CI* confidence interval, *HI* high-intensity training group, *IV* inverse variance, *LI* low-intensity training group, *MI* moderate-intensity training group, *Random* random effects model, *RT* resistance training, *SE* standard error, *SMD* standardized mean difference, *Weight* weight attributed to each study due to its statistical power

Nine studies examined the effects of RT on measures of muscle morphology. An *I*^2^ value of 0 % (*χ*^2^ = 7.18, *df* = 10, *p* = 0.71) is indicative of non-existent heterogeneity, which is why no further sub-analyses were computed (Fig. 3). We pooled weighted mean SMD_bs_ across the nine studies and observed a small effect (mean SMD_bs_ = 0.42; 95 % CI 0.18–0.66) of RT on measures of muscle morphology.

### Methodological Study Quality

Table 2 shows that the quality scores averaged 4.6 ± 1.2 points (range 2–7). This is indicative of low methodological study quality even though only RCTs were included. Three studies [35, 41, 43] were identified that exceeded the pre-determined cut-off score [19] of 6 points or higher.

### Dose–Response Relationships of RT on Measures of Muscle Strength

To improve the generalizability and external validity of our study findings, we combined the results from 25 studies that examined lower/upper extremity muscle strength based on 1RM or MVC tests. Such pooling of data was done to explore the effects of training variables on muscle strength using meta-regression (Table 3). In addition to meta-regression, dose–response relationships were calculated independently using the effect size of characteristics of each training variable (Table 4).

**Table 2:** Physiotherapy Evidence Database (PEDro) scores of the 25 included studies

Authors	Eligibility criteria	Random allocation	Concealed allocation	Baseline comparability	Blind subjects	Blind therapists	Blind assessor	Adequate follow-up dropout <15 %	Intention-to-treat analysis	Between-group comparisons	Point estimates and variability	Score
Beneka et al. [31]	−	+	−	+	−	−	−	+	−	+	+	5
Charette et al. [92]	+	+	−	+	−	−	−	−	−	+	+	4
Daly et al. [93]	−	+	+	−	−	−	+	+	−	+	−	5
DeBeliso et al. [94]	−	+	−	+	−	−	−	−	−	+	+	4
Fatouros et al. [95]	−	+	−	+	−	−	−	+	−	+	+	5
Fatouros et al. [33]	−	+	−	+	−	−	−	+	−	+	+	5
Granacher et al. [36]	−	+	−	−	−	−	−	−	−	−	+	2
Henwood and Taaffe [40]	+	+	−	+	−	−	−	+	−	+	+	5
Hortobagyi et al. [34]	−	+	−	+	−	−	−	+	−	+	+	5
Hunter et al. [96]	+	+	−	+	−	−	−	+	−	+	+	5
Judge et al. [101]	+	+	−	+	−	−	+	+	+	+	+	7
Kalapotharakos et al. [71]	−	+	−	+	−	−	−	−	−	+	+	4
Kalapotharakos et al. [35]	−	+	−	+	−	−	+	+	−	+	+	6
Lovell et al. [97]	+	+	−	+	−	−	−	+	−	+	+	5
Miszko et al. [98]	−	+	−	−	−	−	−	−	−	+	+	3
Morse et al. [99]	−	+	−	−	−	−	−	−	−	+	+	3
Pinto et al. [41]	+	+	−	+	−	−	+	+	+	+	+	7
Pyka et al. [39]	−	+	−	+	−	−	−	+	−	+	+	5
Raso et al. [42]	+	+	−	+	−	−	−	−	−	+	+	4
Reeves et al. [37]	−	+	−	+	−	−	−	−	−	−	+	3
Rhodes et al. [100]	+	+	−	+	−	−	−	+	−	+	+	5
Strasser et al. [72]	+	+	−	+	−	−	−	−	−	+	+	4
Tracy et al. [38]	−	+	−	+	−	−	+	−	−	+	+	5
Vincent et al. [73]	−	+	−	+	−	−	−	+	−	+	+	5
Vincent et al. [32]	−	+	−	+	−	−	−	−	−	+	+	4
Mean score												4.6

+ indicates a “yes” score, − indicates a “no” score

#### Meta-Regression Analysis for Training Variables of Muscle Strength

Table 3 shows the results of the meta-regression for three subcategories: training volume, training intensity, and rest. Concerning training volume, only training period predicted (*p* = 0.04) the effects of RT on muscle strength. In the subcategory training intensity, the best predictors for the explanation of effects of RT on muscle strength were intensity (*p* < 0.05) and time under tension (*p* < 0.01). The mode of muscle action (i.e., isometric, concentric, eccentric) did not influence the effects of RT (*p* = 0.41–0.91). Rest in between sets (*p* = 0.06, trend) and in between repetitions did not predict strength gains.

**Table 3:** Meta-regression for training variables of different subcategories to predict RT effects on muscle strength

	Coefficient	Standard error	95 % lower CI	95 % upper CI	Z value	*P* value
Training volume						
Training period	0.0316	0.0155	0.0012	0.0619	2.04	0.04
Training frequency	0.0900	0.3315	−0.5598	0.7397	0.27	0.79
Number of sets	0.1142	0.1810	−0.2406	0.4690	0.63	0.53
Number of repetitions per set	0.0219	0.0585	−0.0927	0.1366	0.37	0.71
Training intensity						
Training intensity	0.0182	0.0052	0.0084	0.0288	3.57	0.01
Time under tension	0.3154	0.1094	0.1010	0.5297	2.88	0.01
Rest						
Rest in between sets	0.0095	0.0051	−0.0006	0.0196	1.85	0.06
Rest in between repetitions	0.1600	0.2255	−0.282	0.6019	0.71	0.48

*CI* confidence interval, *RT* resistance training

#### Training Period

**Fig. 4:**
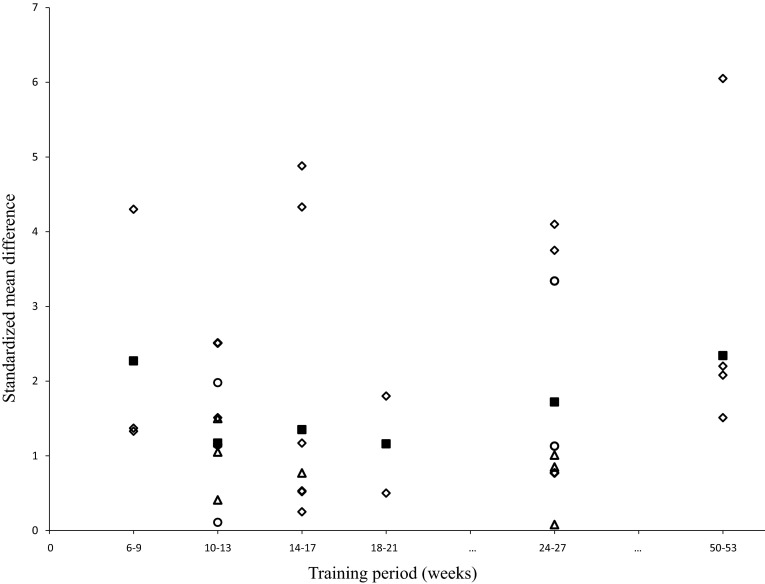
Dose-response relationships for training period and measures of muscle strength following resistance training. Each *unfilled symbol* illustrates the SMD_bs_ per single study. *Filled black squares* represent the weighted mean SMD_bs_ of all studies. *Diamonds*, *circles*, and *triangles* symbolize high-, moderate-, and low-intensity resistance training groups, respectively. *SMD*_*bs*_ between-subject standardized mean difference

On average, the training period in the 25 studies lasted 21.2 weeks (range 6–52 weeks). Figure 4 demonstrates dose–response relationships for the training variable “training period”. Mean SMD_bs_ amounted to 1.57 (95 % CI 1.20–1.94; *I*^2^ = 81 %, *χ*^2^ = 163.10, *df* = 32, *p* < 0.01). The longest training intervention lasted 50–53 weeks and revealed the largest mean SMD_bs_, with a value of 2.34.

#### Training Frequency

Twenty-five studies were included in this sub-analysis, and the mean training frequency was 2.9 sessions per week, with a mean SMD_bs_ of 1.57 (range two to three sessions per week; 95 % CI 1.20–1.94; *I*^2^ = 79 %, *χ*^2^ = 163.10, *df* = 32, *p* < 0.01). That is, two and three training sessions per week produced large effects on measures of muscle strength, with mean SMD_bs_ of 2.13 (two sessions) and 1.49 (three sessions).

#### Number of Sets and Repetitions

In the 25 studies included in this sub-analysis, the number of sets per exercise averaged 2.9 (range one to five sets) and the number of repetitions per set averaged 10.0 (range five to 16 repetitions). Mean SMD_bs_ for number of sets and repetitions per exercise were 1.57 (95 % CI 1.20–1.94; *I*^2^ = 80 %, *χ*^2^ = 163.10, *df* = 32, *p* < 0.001) and 1.61 (95 % CI 1.22–1.99; *I*^2^ = 81 %, *χ*^2^ = 161.71, *df* = 31, *p* < 0.01), indicative of large effects. Two to three sets per exercise (mean SMD_bs_ = 2.99) and seven to nine repetitions (mean SMD_bs_ = 1.98) resulted in the largest improvements in muscle strength.

#### Training Intensity

**Fig. 5:**
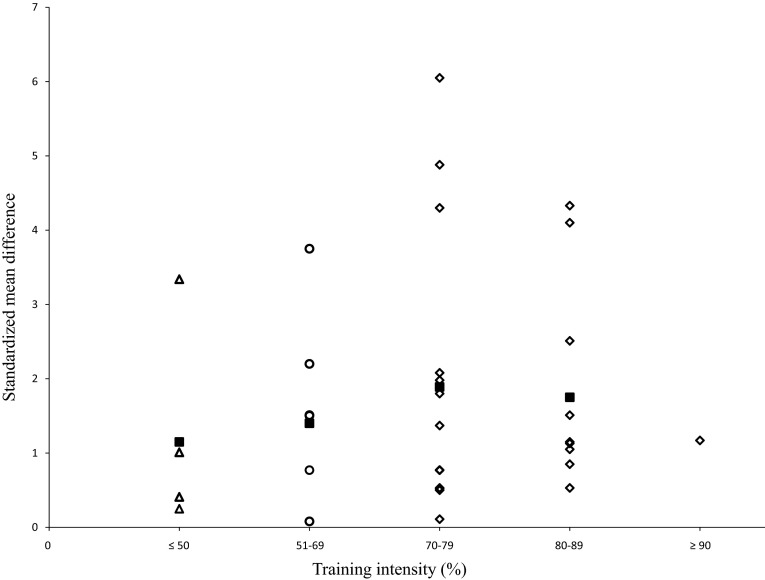
Dose-response relationships for training intensity and measures of muscle strength following resistance training. Each *unfilled symbol* illustrates the SMD_bs_ per single study. *Filled black squares* represent the weighted mean SMD_bs_ of all studies. *Diamonds*, *circles*, and *triangles* symbolize high-, moderate-, and low-intensity resistance training groups, respectively. *SMD*_*bs*_ between-subject standardized mean difference

Twenty-four studies were included in this sub-analysis, and training intensity was classified as high (≥70 % 1RM), moderate (51 % ≥ 1RM ≤ 69 %), and low (≤50 % 1RM) [16]. The sub-analysis revealed a mean intensity of 69 % of the 1RM (range 40–90 % 1RM) across studies. Figure 5 illustrates dose–response relationships for training intensity, with a mean SMD_bs_ of 1.63 (95 % CI 1.21–2.05; *I*^2^ = 82 %, *χ*^2^ = 157.81, *df* = 28, *p* < 0.01). The largest effects on measures of muscle strength were found for intensities of 70–79 % of the 1RM (mean SMD_bs_ = 1.89).

#### Time Under Tension per Repetition

**Fig. 6:**
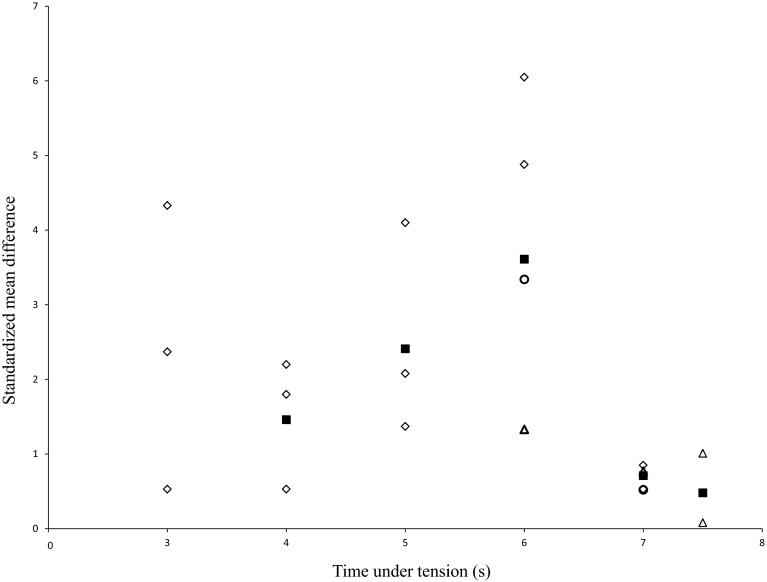
Dose-response relationships for total time under tension and measures of muscle strength following resistance training. Each *unfilled symbol* illustrates the SMD_bs_ per single study. *Filled black squares* represent the weighted mean SMD_bs_ of all studies. *Diamonds*, *circles*, and *triangles* symbolize high-, moderate-, and low-intensity resistance training groups, respectively. *SMD*_*bs*_ between-subject standardized mean difference

Time under tension is an important variable to induce adaptations in muscle strength and morphology [17]. In 14 studies, the total time under tension averaged 5.7 s per repetition (range 3–7.5 s; mean SMD_bs_ = 1.60; 95 % CI 1.09–2.10; *I*^2^ = 82 %, *χ*^2^ = 102.65, *df* = 18, *p* < 0.01). The largest effect was shown for 6 s, with a mean SMD_bs_ of 3.61. Figure 6 shows the dose–response relationships for the training variable “time under tension”. In addition, the mean time under tension was 2.3 s for isometric (range 2–2.5 s; SMD_bs_ = 2.48; 95 % CI 1.36–3.32; *I*^2^ = 83 %, *χ*^2^ = 47.19, *df* = 8, *p* < 0.01), 2.2 s for concentric (range 1.5–4.0 s; SMD_bs_ = 2.18; 95 % CI 1.26–2.54; *I*^2^ = 84 %, *χ*^2^ = 101.94, *df* = 16, *p* < 0.01), and 2.5 s for eccentric actions (range 1.5–3.5 s; SMD_bs_ = 2.28; 95 % CI 1.36–2.79; *I*^2^ = 87 %, *χ*^2^ = 123.06, *df* = 16, *p* < 0.01). During the isometric mode, a time under tension of 2.0 s with a mean SMD_bs_ of 2.70 appears most effective. In the concentric and eccentric modes, times under tension of 2.5 s (mean SMD_bs_ = 3.44) and 3.0 s (mean SMD_bs_ = 2.98) seem to be most effective.

#### Rest Time (Rest in Between Sets and Repetitions)

**Fig. 7:**
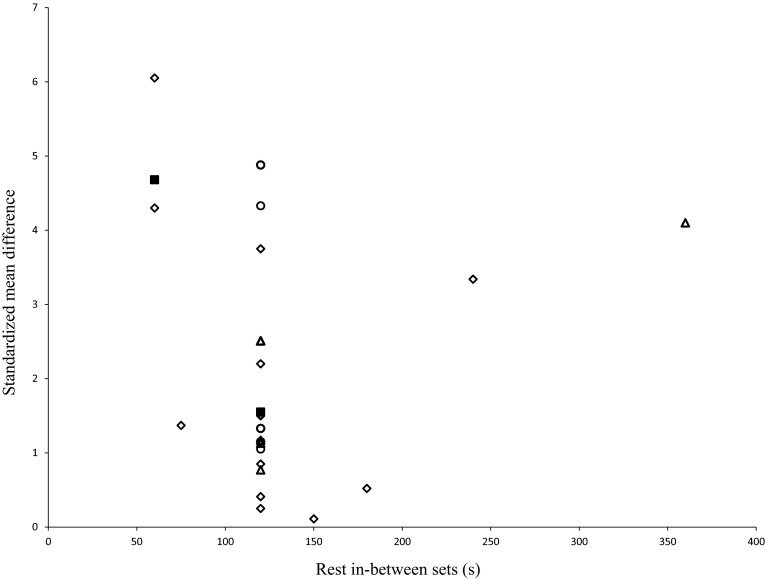
Dose-response relationships for rest in between sets and measures of muscle strength following resistance training. Each *unfilled symbol* illustrates the SMD_bs_ per single study. *Filled black squares* represent the weighted mean SMD_bs_ of all studies. *Diamonds*, *circles*, and *triangles* symbolize high-, moderate-, and low-intensity resistance training groups, respectively. *SMD*_*bs*_ between-subject standardized mean difference

Based on data from 17 studies, we computed dose–response relationships regarding rest time between sets and/or repetitions. The mean rest time between sets was 132 s (range 60–360 s; mean SMD_bs_ = 1.87; 95 % CI 1.35–2.38; *I*^2^ = 84 %, *χ*^2^ = 138.61, *df* = 22, *p* < 0.01), and between repetitions (five studies) it was 3.9 s (range 1.5–5 s; mean SMD_bs_ = 2.24; 95 % CI 1.52–2.31; *I*^2^ = 83 %, *χ*^2^ = 47.19, *df* = 8, *p* < 0.01). Figure 7 shows the dose–response relationships for the training variable “rest in between sets”. Eleven out of 17 studies used 120 s of rest in between sets, resulting in a mean SMD_bs_ of 1.57. With reference to the results of two studies [39, 40], a rest in between sets of 60 s appears to be most effective to increase muscle strength (mean SMD_bs_ = 4.68) (Fig. 7). A rest time between repetitions of 4.0 s seems to be most effective, coupled with a mean SMD_bs_ of 3.72.

### Dose–Response Relationships of RT on Measures of Muscle Morphology

#### Meta-Regression Analyses for Training Variables of Muscle Morphology

Due to the low number of studies, we performed meta-regression only for the subcategory “training volume”. The regression analysis revealed that no variable within the training volume subcategory (i.e., period, frequency, number of sets, number of repetitions) produced significant effects (*p* = 0.52–0.94) on measures of muscle morphology.

#### Training Period

Pooled data from nine studies revealed a mean training period of 24.0 weeks (range 6–52 weeks), with a mean SMD_bs_ of 0.42 (95 % CI 0.18–0.66; *I*^2^ = 0 %, *χ*^2^ = 7.18, *df* = 10, *p* = 0.71). With reference to the results of one study [41], a training period of 6 weeks appeared to be most effective to improve measures of muscle morphology, with an SMD_bs_ of 0.66. Of note, the results of the two studies that used 50–53 weeks as a training period showed a slightly lower effect on measures of muscle morphology (mean SMD_bs_ = 0.59).

#### Training Frequency

Our sub-analysis included nine studies and revealed a mean training frequency of 2.9 training sessions per week (range two to three sessions per week), with a mean SMD_bs_ of 0.42 (95 % CI 0.18–0.66; *I*^2^ = 0 %, *χ*^2^ = 7.18, *df* = 10, *p* = 0.71). The results of one study [41] suggested the largest improvement in measures of muscle morphology with two (SMD_bs_ = 0.66) compared with three sessions per week (mean SMD_bs_ = 0.38). Of note, eight out of nine studies examined the effects of three training sessions per week.

#### Number of Sets and Repetitions

Based on nine studies, the average number of sets per exercise was 2.3 (range one to three sets). On average, 10.6 repetitions (range eight to 16 repetitions) were performed per set. The mean SMD_bs_ for number of sets as well as repetitions per exercise was 0.54 (95 % CI 0.30–0.78; *I*^2^ = 0 %, *χ*^2^ = 7.25, *df* = 10, *p* = 0.70) and 0.42 (95 % CI −0.32–0.90; *I*^2^ = 0 %, *χ*^2^ = 0.08, *df* = 1, *p* = 0.77), indicative of moderate and small effects, respectively. Two to three sets per exercise (mean SMD_bs_ including two studies = 0.78) and seven to nine repetitions (mean SMD_bs_ = 0.49; six studies) resulted in the largest improvements in measures of muscle morphology based on findings of more than one study. One study conducting RT with 16–18 repetitions per set reported an SMD_bs_ of 0.66.

#### Training Intensity

Eight studies that reported training intensities were classified as high (≥70 % 1RM), moderate (51 % ≥ 1RM ≤ 69 %), and low (≤50 % 1RM) [16]. Mean intensity across studies was 71 % of the 1RM (range 50–80 % of 1RM), with a mean SMD_bs_ of 0.38 (95 % CI 0.13–0.64; *I*^2^ = 0 %, *χ*^2^ = 6.61, *df* = 9, *p* = 0.68). Exercise at a moderate intensity between 51 and 60 % of the 1RM produced the greatest effects on measures of muscle morphology, with a mean SMD_bs_ of 0.43 (four studies). One study showed the same effect (SMD_bs_ = 0.43) on muscle volume using an intensity of 70–79 % of 1RM.

#### Time Under Tension per Repetition

Based on two studies, the total time under tension averaged 5.3 s, with a mean SMD_bs_ of 0.31 (range 4–6 s; 95 % CI −0.18 to 0.80; *I*^2^ = 0 %, *χ*^2^ = 0.10, *df* = 2, *p* = 0.95). The largest effect occurred at 6 s, with a mean SMD_bs_ of 0.36 (one study). Considering specific muscle action modes, only one study [35] reported time under tension during isometric muscle actions and two studies [35, 42] reported time under tension for concentric and eccentric muscle actions. The mean time under tension was 2.0 s for the isometric mode (SMD_bs_ = 0.36; 95 % CI 1.13–4.27; *I*^2^ = 75 %, *χ*^2^ = 7.98, *df* = 2, *p* = 0.02), 1.8 s for the concentric mode (range 1.5–2 s; SMD_bs_ = 0.31; 95 % CI −0.18 to 0.80; *I*^2^ = 0 %, *χ*^2^ = 0.10, *df* = 2, *p* = 0.95), and 2.2 s for the eccentric mode (SMD_bs_ = 0.31; 95 % CI −0.18 to 0.80; *I*^2^ = 0 %, *χ*^2^ = 0.10, *df* = 2, *p* = 0.95). The most effective time under tension appears to be 2.0 s for isometric, concentric, and eccentric muscle actions (SMD_bs_ = 0.36; one study), respectively.

#### Rest Time (Rest in Between Sets and Repetitions)

In each of the six studies, the mean rest time was 120 s between sets. Only one study [35] provided detailed information regarding rest time between repetitions (2.5 s). The mean SMD_bs_ was 0.30 for rest in between sets (95 % CI 0.04–0.57; *I*^2^ = 0 %, *χ*^2^ = 1.74, *df* = 7, *p* = 0.97) and 0.36 for rest in between repetitions (95 % CI −0.24 to 0.96; *I*^2^ = 0 %, *χ*^2^ = 0.00, *df* = 1, *p* = 0.95).

## Discussion

To the best of our knowledge, this is the first systematic literature review and meta-analysis that provides an integrated overview of the general effectiveness of RT on measures of muscle strength and morphology in healthy old adults. The results from the 25 eligible RCTs suggest a large and systematic training effect of RT on muscle strength (Fig. 2) and a small effect on measures of muscle morphology (Fig. 3). We also performed a meta-regression analysis to determine how such training variables as volume, intensity and rest modify the RT effects on measures of muscle strength and morphology. Additional dose–response relationships of each training variable were computed independently from the other training variables (Table 4). Moreover, we discuss the findings with reference to the relevant literature concerning the general effects and dose–response relationships following RT in healthy old adults. If no age-group specific information was available in the literature, we extended our search and discussion to findings regarding the effects of RT in healthy young adults.

**Table 4:** Training variables with largest mean SMD_bs_

Training variables	Measures of muscle strength		Measures of muscle morphology	
	Highest value	Mean SMD_bs_	Highest value	Mean SMD_bs_
Training period [weeks]	50–53	2.34	50–53	0.59^a^
Training frequency [sessions per week]	2	2.13	3	0.38
Number of sets per exercise	2–3	2.99	2–3	0.78^a^
Number of repetitions [per set]	7–9	1.98	7–9	0.49
Training intensity [% of 1RM]	70–79	1.89	51–69	0.43
Time under tension (total) [s]	6.0	3.61	6	0.36^a^
Time under tension (isometric mode) [s]	2.0	2.70^a^	2.0	0.36^a^
Time under tension (concentric mode) [s]	2.5	3.44	2.0	0.36^a^
Time under tension (eccentric mode) [s]	3.0	2.98	2.0	0.36^a^
Rest in between sets [s]	60	4.68^a^	120	0.30
Rest in between repetitions [s]	4	3.72^a^	2.5	0.36^a^

The content of this table is based on individual training variables with no respect for interaction between training variables

*SMD*_*bs*_ between-subject standardized mean difference, *1RM* one-repetition maximum

^a^Based on less than three studies

### Effects of RT on Measures of Muscle Strength and Morphology in Healthy Old Adults

In healthy old adults, RT improved muscle strength substantially (13–90 %; 25 studies) and measures of muscle morphology to a smaller extent (1–21 %; nine studies). The results seem to suggest that the various forms of RT reviewed here have a greater potential to improve healthy old adults’ ability to generate maximal voluntary force compared with the potential to improve measures of muscle morphology (mean SMD_bs_ = 1.57 vs. 0.42). These findings are in line with the results of two meta-analyses, which examined the effects of RT on muscle strength [12] and size [44] in healthy as well as frail and/or disabled middle-aged and/or old adults (range 50–95 years) and reported increases in muscle strength and size of 24–33 % and 1.5–16 %, respectively [13–16]. Recent imaging, magnetic brain stimulation, and peripheral nerve stimulation studies seem to lend support to the emerging hypothesis that life-long RT could be an important non-pharmaceutical intervention to slow the age-related neural dysfunction through which muscle strength loss can be reduced [45–54]. This prediction is corroborated by in vitro evidence suggesting that age and disuse do not affect intrinsic upper- and lower-limb skeletal muscle function even in the oldest-old. While age does affect in vivo whole muscle function, which is exacerbated by disuse [55], RT could effectively counteract the age-related strength loss. The effectiveness of RT was investigated by the present and several previous reviews [12–16]. Further, Delmonico et al. [2] conducted a 5-year longitudinal study with well-functioning men and women (*N* = 1678) between the ages of 70 and 79 years at baseline and measured knee extensor torque using an isokinetic device and mid-femur CSA using computer tomography at the beginning of the study and after 6 years. It was found that decreases in isokinetic leg muscle torque were two to five times greater than losses in CSA with aging and that the change in quadriceps muscle area only explains about 6–8 % of the between-subject variability in the change in knee extensor torque. This implies that the loss in muscle strength with age (dynapenia) is more related to impairments in neural activation and/or reductions in the intrinsic force-generating capacity of skeletal muscle [3]. Based on these findings, it seems plausible to argue that primarily neural adaptations account for training induced improvements in muscle strength, with improvements in measures of muscle morphology playing a minor role, particularly during the early phase of RT [56]. This may explain the observed larger gains in muscle strength compared with measures of muscle morphology [2, 7].

Despite the large effect of RT on muscle strength, there was still considerable variation in the magnitude of adaptations between studies. Methodological issues may also contribute to the large variability. For example, the magnitude of response varies between body regions (upper vs. lower limbs) or muscle groups. Adaptations to RT can be highly specific, as training-induced changes in CSA can differ between vastus lateralis and vastus medialis and can also be muscle-length specific [57]. Another factor contributing to the large variation in the response to RT is the age of the subjects, which ranged widely, between 60 and 90 years. Spontaneous physical activity is much higher for seniors at age 65 vs. 85, with some older individuals making as few as 100–200 steps per day [58]. The observations from a large cross-sectional study that in some healthy old cohorts there could be accelerated muscle strength loss even as early as age 60–69 just further strengthen the argument for prescribing RT for old adults aging healthily [1].

### Dose–Response Relationships of RT to Increase Muscle Strength

The previous section established a large overall effect of RT on maximal voluntary strength in healthy old adults. We further performed meta-regression to identify training variables that affected strength gains after conducting RT. To specify the characteristic of each training variable with the largest effect on muscle strength, we conducted additional analyses of independently computed dose–response relationships.

#### Training Volume (Period, Frequency, Number of Sets, Number of Repetitions)

Of the four training variables within training volume, meta-regression identified training period only to have a significant effect on muscle strength. The longest training period produced the largest increases in voluntary muscle strength (mean SMD_bs_ = 2.34; 50–53 weeks). This result is based on only four studies, as in the majority of the studies the intervention duration ranged from 6 to 26 weeks. Curiously, RT as short as 6–9 weeks was only slightly less effective than RT of 50–53 weeks to improve muscle strength (mean SMD_bs_ = 2.27; two studies). This observation suggests that RT is a suitable intervention to combat weakness in healthy old adults because the nervous system exhibits a rapid responsiveness to mechanical overload [4, 30, 49, 51, 59]. In agreement with our findings, a current meta-analysis that included 15 studies confirmed the outcome of the general analysis that “training period” is the only significant variable (*p* < 0.01) to improve muscle strength based on results of meta-regression [15]. These authors reported that long (24–52 weeks) versus short training periods (8–18 weeks) are more effective. In addition, Kennis et al. [60] investigated detraining effects following 1 year of RT on different variables of muscle strength in old adults (60–80 years). After 7 years of detraining, initially strength-trained participants still exhibited improved muscle strength characteristics compared with the control group. However, the authors pointed out that RT cannot attenuate the age-related decline in muscle strength and therefore suggested the application of lifelong RT. These findings are in accordance with ACSM recommendations [61].

In contrast to the results of meta-regression, additional analyses of dose–response relationships indicated large differences between two training sessions per week (mean SMD_bs_ = 2.13) and three training sessions per week (mean SMD_bs_ = 1.49). Because studies that administered two sessions per week were also of short duration (6–9 weeks), learning effects and neuronal adaptions must have contributed strongly to the effects associated with two versus three sessions per week [4, 30, 49, 51, 59]. In support of our meta-regression data, DiFrancisco-Donoghue et al. [62] reported similar increases in muscle strength after 9-week-long programs consisting of one and two weekly sessions in healthy old adults age 65–79. Furthermore, Taaffe et al. [63] conducted a 24-week RT intervention with three different training frequencies (one to three sessions per week) in old adults aged 65–79 years. The authors concluded that a weekly or biweekly RT is equally effective to enhance muscle strength as compared with three sessions per week. Of note, our findings must be interpreted with caution because the range of training frequencies was narrow (two to three sessions per week). Finally, the current meta-analysis confirms the conclusion reached by expert opinion in the ACSM position stand that recommended RT frequencies of at least two sessions per week [61].

Our analyses revealed little or no effect of the training variables “number of sets per exercise” and “number of repetitions per set” on strength gains. The additional analyses of dose–response relationships of the number of sets per exercise revealed an inverse U-shape, with the largest effect (mean SMD_bs_ = 2.99) being prevalent in RT protocols that applied two to three sets. However, it seems that there is no difference between single versus multiple sets in short-term RT (6 weeks) in old adults [64]. Moreover, these results suggested that during the early phase of RT, number of sets was not the primary variable responsible for increases in muscle strength and thickness in old adults [64]. In addition, “number of sets” appears not to result in neural adaptations because no differences were found in electromyography activation of quadriceps muscles between groups of old women (60–74 years) that trained using single or multiple sets [64]. But although the musculoskeletal system is adapted through the stimulus of a single set to failure, multiple sets appear to be required to add continued strength gains [65]. Multiple versus single number of sets seemingly has a higher impact on muscle strength in combination with longer training periods. In this context, Radaelli et al. [66] examined the effects of one set, three sets, and five sets of RT applied over a period of 6 months (three sessions per week) on measures of upper- and lower-limb muscle strength and muscle thickness in young untrained men age 24 years. Multiple versus single sets improved muscle strength and muscle thickness particularly of the upper body more effectively, especially with five sets of RT. In addition, two non-RCTs investigated the impact of one set or three sets per exercise on measures of muscle strength in old adults aged 60–80 years [67, 68]. Only the study examining a longer training period (20 vs. 12 weeks) found a significant effect of three-set versus one-set training on peak torque and maximum voluntary contraction of the knee extensors in elderly subjects aged 65–78 years [68]. Together, there is a paucity of data from high-quality RCTs concerning the effects of training frequency on muscle strength, especially in the elderly.

Finally, concerning the training variable “number of repetitions”, the largest effects in strength gains occurred when old adults used seven to nine repetitions per set (mean SMD_bs_ = 1.98). Despite that the “number of repetitions” within a set in RT could provide a distinct physiological stimulus for strength gains—with lower repetitions predicted to be more effective [69]—our systematic search identified no study that specifically examined the effects of different repetitions per set on variables of muscle strength. This can most likely be explained by the fact that the variable “number of repetitions” is often used as an indicator of training intensity, which is why previous research efforts focused on “training intensity” rather than “number of repetitions”. In fact, it has been reported that a given percentage of the 1RM determines the realized number of repetitions within a set until failure [15]. For that reason, lower repetitions resulted in higher training intensity that induced greater acute neuromuscular fatigue accompanied by greater hormonal responses [70].

#### Training Intensity (Intensity, Time Under Tension)

In support of the meta-regression results that training intensity (*p* < 0.01) predicted the effects of RT on muscle strength, the largest effect of RT (intensity mean SMD_bs_ = 1.89) on 1RM strength occurred when strength training intensity was set at 70–79 % of 1RM (range 40–90 % 1RM, Fig. 5). Our systematic search identified six studies that directly compared RT protocols of different intensities [31–35]. This analysis showed that high-intensity RT produced the largest effects on muscle strength in comparison to moderate- (high vs. moderate mean SMD_bs_ = 0.60) or low-intensity (high vs. low mean SMD_bs_ = 0.88) training regimes. Also, moderate-intensity RT produced a larger effect on muscle strength compared with low-intensity RT (moderate vs. low mean SMD_bs_ = 0.93). The effects of moderate- and low-intensity RT compared with a passive control group had a mean SMD_bs_ of 1.75 and 1.02 in favor of RT [31, 33–35, 42, 71–73].

Previous meta-analyses suggested similar effects of high-intensity RT (≥70 % 1RM) compared with moderate- [e.g., mean SMD_bs_ (high vs. moderate) = 0.62] and low-intensity [e.g., mean SMD_bs_ (high vs. low) = 0.88] RTs [12, 14, 15] on muscle strength in healthy old adults. These findings are in accordance with the ACSM position stand that states higher intensities result in greater strength gain in old adults [61]. Nevertheless, recent reviews rated the importance of training intensity as a training variable to be of minor relevance if no other training variables (i.e., time under tension, rest time) were considered [15, 74]. Training intensity defined as the individual percentage of 1RM, appears not to be as sensitive as the rate of perceived exertion using, for instance, the OMNI resistance exercise scale [75]. In other words, the number of repetitions conducted at a given percentage of 1RM differs inter-individually because of training status, and intra-individually because of the muscle groups trained [75]. Therefore, the 1RM represents a method to regulate training intensity that should always be combined with information about the time under tension [17, 74].

Total time under tension had a strong effect (*p* < 0.01) on strength gains, with 6 s per repetition producing the largest effect size (mean SMD_bs_ = 3.61; 14 studies, range 3–7.5 s). The time under tension is an important variable for mechano-biological adaptations, because different times under tension affect different metabolic changes as well as motor unit (MU) recruitment and MU firing rates occurring during RT [17]. Furthermore, temporal distribution of isometric, concentric, and eccentric muscle action per repetition seemed to be also important [17]. However, the mode of muscle action (isometric, concentric, eccentric) had no effect on strength gains (*p* = 0.41–0.91). Our search identified 14 studies that reported information on muscle action-specific time under tension per repetition during RT (isometric: four studies, range 2.0–2.5 s; concentric: 14 studies, range 1.5–4 s; eccentric: 13 studies, range 1.5–3.5 s). The most effective time under tension amounted to 2.0 s (mean SMD_bs_ = 2.70), 2.5 s (mean SMD_bs_ = 3.44), and 3.0 s (mean SMD_bs_ = 2.98) for isometric, concentric, and eccentric muscle actions, respectively. But to the best of our knowledge, there is no study that compared the effects of contraction duration on strength gains. The meta-analysis of Roig et al. [76] allows us at least some insight into muscle action-specific adaptive processes in healthy adults aged 18–65 years. These authors stated that separate eccentric muscle actions produce larger gains in muscle strength and morphology compared with concentric muscle actions. However, these findings have to be interpreted with caution because in several cases, isotonic RT is applied, which consists of concentric and eccentric muscle actions, so that information on muscle action-specific time under tension is needed. It has previously been hypothesized that a longer eccentric phase results in improved training efficiency because eccentric loads affect the protein synthesis and muscle activation and thus muscle hypertrophy and strength [77, 78]. The results concerning time under tension are limited by the low number of studies and by a lack of direct determination of the muscle action duration effects on strength gains. For example, no study has performed RT with longer contraction duration than 7.5 s per muscle action. Based on our and previous findings [17], we recommend that authors report time under tension, measured or estimated, as this seems an important variable underlying gains in muscle strength and muscle morphology.

#### Rest (Rest in Between Sets and Repetitions)

Meta-regression revealed that rest between sets (*p* = 0.06) and repetitions did not modify the effects of RT on muscle strength. Of the two specific studies that examined dose–response relationship with respect to rest in between sets, one using 60 s produced the largest mean SMD_bs_ of 4.68 in healthy old adults. The overall analysis is limited by a uniform use of 120-s rest in between sets, resulting in a mean SMD_bs_ of 1.57 (Fig. 7). The recent study of Villanueva et al. [79] investigated the effects of short (60-s) vs. long (240-s) rest intervals between sets on muscle strength and lean body mass after 8-week RT (3×/week, 2–3 sets, 4–6RM) in 22 old men aged 66 years. The findings revealed that short rest intervals between sets resulted in significant greater increases in leg press 1RM (*p* < 0.001) and in lean body mass (*p* = 0.001). Moreover, it is suggested that less rest times produced greater levels of fatigue, providing a stimulus which resulted in increases in muscle strength [17, 79, 80]. Furthermore, Willardson [81] hypothesized in a narrative review that shorter rests in between sets are associated with a more prominent hypertrophic effect. In addition, there is information in the literature stating that the duration of rest in between sets has to be configured to the training goal. Based on different metabolic and hormonal loads, a narrative review suggested that rest in between sets of 180–300 s is suitable for improvements in maximal strength, 1–2 min for gains in hypertrophy and 30–60 s for improvements in muscle endurance [30, 82].

The training variable “rest time between repetitions” was computed independently to elucidate dose–response relationships, and the results indicated that a 4.0-s rest in between repetitions seems to be most effective to increase muscle strength (mean SMD_bs_ = 3.72). However, this finding is preliminary because it is based on one study with three training groups only. Nevertheless, the variable “rest in between repetitions” seems to be a significant mechano-biological determinant of myocellular oxygen homeostasis [17]. Therefore, it needs to be specified in RT protocols.

None of the five included studies reported the reason for the duration of rest used between repetitions. Furthermore, no other study compared the effects of in between repetitions rest on strength gains at any age. Basically, the efficiency of RT (i.e., duration of a single training session) is influenced by the amount of rest in between repetitions. However, longer rest times between repetitions prolong the time of a single training session and may thus make training less efficient. On the other hand, longer rest times between repetitions might be particularly beneficial in old adults because acute deteriorations in postural control were reported following one bout of high-intensity RT exercise (four sets) [83]. Longer rest times during RT exercises may affect postural control to a lesser extent by reducing the acute risk of falling during training [83]. This review provided for the first time information on how to effectively implement rest in between repetitions in RT protocols for old adults. Based on the low number of studies (five studies) and the results of meta-regression, these findings should be interpreted with caution and further studies are needed.

### Dose–Response Relationships of RT to Improve Measures of Muscle Morphology

To the best of our knowledge, no systematic review or meta-analysis has examined whether changes in muscle morphology would scale according to RT dose in healthy old adults. Due to a low number of studies, we could only examine the effects of training volume on measures of muscle morphology. We found that variation in the volume of RT had no effect on measures of muscle morphology. A training period of 6 weeks and using 16–18 repetitions per set during RT is ineffective for muscle hypertrophy. We interpret this unexpected result [41] as an abnormality caused by the choice of unusual training variables (6 weeks of training; 16–18 repetitions per set), producing an SMD_bs_ of 0.66 [41]. Nevertheless, a cumulative analysis of the remainder of the studies revealed the following specific effects on healthy old adults’ muscle morphology when conducting RT with a training period of 50–53 weeks (mean SMD_bs_ = 0.59), a training frequency of three sessions per week (mean SMD_bs_ = 0.38), a training volume of two to three sets per exercise (mean SMD_bs_ = 0.78), seven to nine repetitions per set (mean SMD_bs_ = 0.49), a training intensity of 51–69 % of the 1RM (mean SMD_bs_ = 0.43), a total time under tension of 6 s (mean SMD_bs_ = 0.36), a time under tension of 2.0 s for isometric, concentric, and eccentric muscle actions (mean SMD_bs_ = 0.36 each), respectively, a rest between sets of 120 s (mean SMD_bs_ = 0.30), and a rest between repetitions of 2.5 s (mean SMD_bs_ = 0.36). In general, our findings agree with results reported previously [13, 84, 85]. The meta-analysis of Peterson et al. [13] suggested that RT with a mean training period of 21 weeks (three training sessions per week), an intensity of 75 % of the 1RM, two to three sets and ten repetitions with a 110-s rest in between sets was effective to significantly increase lean body mass in old adults (weighted pooled estimate 1.1 kg; 95 % CI 0.9–1.2). The narrative reviews of Mayer et al. [84] and Petrella and Chudyk [85] also illustrated dosage of training variables to prevent the loss of muscle mass. These authors recommended the following RT variables to prevent the loss of muscle mass in old age: training period of 8–12 weeks, three training sessions per week, training intensities of 60–80 % of the 1RM, three to four sets and eight to 12 repetitions per exercise. These recommendations are consistent with the results of the present meta-analysis. However, we consider our findings preliminary with regard to the effects of RT on measures of muscle morphology because our systematic search identified only nine eligible studies for inclusion in our quantitative sub-analyses and meta-regression could not be performed for all subcategories.

### Limitations and Strengths of this Review

Even though the present review has identified the numerical characteristics of the dose–response relationships, it is a major limitation that such analyses fail to provide insights into the physiological stimulus for increasing old adults’ muscle strength and muscle size. This is a particularly relevant issue because the number of theories concerning the stimulus for strength gains involves fatigue [80], total work [34, 59, 86], hypoxia [87, 88], and time under tension [89] and these factors are often also cited as concurrently acting as stimulus for muscle hypertrophy [3, 90].

The ultimate aim was to establish a possible combination of a set of RT variables that provides an effective training stimulus for slowing age-related muscle strength and muscle mass loss. To investigate the effects of training variables on muscle strength and morphology, subcategories were created on the basis of best applicability for practitioners and clinicians. Afterwards, a meta-regression was performed to find best predictors for effects of RT on measures of muscle strength and muscle morphology. Indeed, we constructed a dose–response relationship from individual RT variables as additional analyses. The variables may be most effective in improving measures of muscle strength and morphology, but it is unclear if the interaction between the so-specified variables would still remain ‘optimal’. We recognize the limitation that our results may not represent one such general dose–response relationship. Modeling of training variables can, however, address this issue; holding a set of RT variables constant while changing the effects of one specific variable could determine the unique effects of each training variable [91]. With regard to training volume, the training effects have to be interpreted with caution because of the difficulty in quantifying training volume if more than one exercise per muscle is performed (e.g., leg press and knee extension/curl). Furthermore, due to the nature of meta-analysis, we focused on those strength outcomes with the highest functional relevance (e.g., dynamic before isometric strength tests). Thus, our findings are outcome specific and cannot necessarily be transferred to different strength outcomes that were not computed in the present study.

The methodological quality of the included studies is rather low because only three out of 25 studies reached the pre-determined cut-off score of 6 points on the PEDro scale that stands for high-quality studies. Of note, possible systematic errors cannot be eliminated because important points (e.g., blinding of subjects or therapists) for internal validity were not considered in all included studies. Furthermore, our findings of effects of RT on measures of muscle morphology have to be considered as preliminary because our systematic search identified only nine studies based on our selected inclusion criteria. Another limitation is that many studies failed to report the training variables. Further, information regarding subject characteristics were often incomplete (e.g., training status, age, health status) and results were inconclusively reported (e.g., means and standard deviation) so that in several cases we were not able to compute SMDs. Future studies should present detailed information and data sets on the investigated cohorts, RT protocols, and study findings. In addition, large heterogeneity was found across studies, which implies a large variability in the tested muscle strength variables (i.e., tests for upper- and lower-extremity muscles) and the investigated cohorts (i.e., large age ranges from 60 to 90 years).

Despite these limitations, this systematic review and meta-analysis is the first to provide an adequate overview of RT effects on measures of muscle strength and muscle morphology in one meta-analysis. The present meta-analysis analyzed sedentary old adults who commenced RT to mitigate the age-related loss of muscle strength and mass. In addition, we were able to extract crucial training variables, such as volume, intensity, and rest, and their dose–response relationships for clinicians and practitioners seeking to implement an effective RT in healthy old adults. Furthermore, we undertook the first attempt to provide dose–response relationships for other important training variables such as time under tension and rest in between sets and repetitions, albeit these were calculated independently of other training variables.

## Conclusion

This systematic literature review and meta-analysis showed that the effects of RT on measures of muscle morphology (mean SMD_bs_ = 0.42) were much smaller compared with the effects on muscle strength (mean SMD_bs_ = 1.57) in healthy old adults. The dose–response relationship analyses showed that training period (50–53 weeks, *p* = 0.04), intensity (70–79 % 1RM, *p* < 0.01), and time under tension (6 s, *p* < 0.01) can significantly and independently modify the RT effects on muscle strength in healthy old adults. Data for other variables were insufficient to draw firm conclusions. It seems that 60 s of rest between sets (*p* = 0.06; two studies), a training frequency of two sessions per week, a training volume of two to three sets per exercise, seven to nine repetitions per set, and 4.0 s between repetitions appear to be the training variables that could have the greatest and most rapid effects on improving maximal voluntary strength in healthy old adults.

RT with the following parameters seems to be effective to improve measures of muscle morphology: a training period of 50–53 weeks, a training frequency of three sessions per week, a training volume of two to three sets per exercise, seven to nine repetitions per set, a training intensity from 51 to 69 % of the 1RM, a total time under tension of 6.0 s, a rest of 120 s between sets and 2.5 s between repetitions. Practitioners, clinicians, and therapists should consult these findings with caution and only as an initial attempt for a comprehensive analysis to characterize RT variables for improving healthy old adults’ muscle morphology. Future studies should particularly focus on the detailed description of training variables (e.g., time under tension) to allow in-depth analysis of dose–response relationships following RT in healthy, mobility limited, and/or frail old adults.
